# Effectiveness of pre-pregnancy lifestyle in preventing gestational diabetes mellitus—a systematic review and meta-analysis of 257,876 pregnancies

**DOI:** 10.1038/s41387-023-00251-5

**Published:** 2023-11-16

**Authors:** Swetha Sampathkumar, Durga Parkhi, Yonas Ghebremichael-Weldeselassie, Nithya Sukumar, Ponnusamy Saravanan

**Affiliations:** 1 Division of Health Sciences, Warwick Medical School, Gibbet Hill, University of Warwick, Warwick, Coventry CV4 7AL UK; 2 School of Mathematics and Statistics, The Open University, Milton Keynes, UK; 3 Academic Department of Diabetes, Endocrinology and Metabolism, George Eliot Hospital, Nuneaton, CV10 7DJ UK

**Keywords:** Gestational diabetes, Lifestyle modification

## Abstract

**Background:**

Gestational Diabetes Mellitus (GDM) is hyperglycaemia first detected during pregnancy. Globally, GDM affects around 1 in 6 live births (up to 1 in 4 in low- and middle-income countries- LMICs), thus, urgent measures are needed to prevent this public health threat.

**Objective:**

To determine the effectiveness of pre-pregnancy lifestyle in preventing GDM.

**Methods:**

We searched MEDLINE, Web of science, Embase and Cochrane central register of controlled trials. Randomized control trials (RCTs), case-control studies, and cohort studies that assessed the effect of pre-pregnancy lifestyle (diet and/or physical activity based) in preventing GDM were included. Random effects model was used to calculate odds ratio (OR) with 95% confidence interval. The Cochrane ROB-2 and the Newcastle-Ottawa Scale were used for assessing the risk of bias. The protocol was registered in PROSPERO (ID: CRD42020189574)

**Results:**

Database search identified 7935 studies, of which 30 studies with 257,876 pregnancies were included. Meta-analysis of the RCTs (*N* = 5; *n* = 2471) in women who received pre-pregnancy lifestyle intervention showed non-significant reduction of the risk of developing GDM (OR 0.76, 95% CI: 0.50–1.17, *p* = 0.21). Meta-analysis of cohort studies showed that women who were physically active pre-pregnancy (*N* = 4; *n* = 23263), those who followed a low carbohydrate/low sugar diet (*N* = 4; *n* = 25739) and those women with higher quality diet scores were 29%, 14% and 28% less likely to develop GDM respectively (OR 0.71, 95% CI: 0.57, 0.88, *p* = 0.002, OR 0.86, 95% CI: 0.68, 1.09, *p* = 0.22 and OR 0.72, 95% CI 0.60–0.87, *p* = 0.0006).

**Conclusion:**

This study highlights that some components of pre-pregnancy lifestyle interventions/exposures such as diet/physical activity-based preparation/counseling, intake of vegetables, fruits, low carbohydrate/low sugar diet, higher quality diet scores and high physical activity can reduce the risk of developing gestational diabetes. Evidence from RCTs globally and the number of studies in LMICs are limited, highlighting the need for carefully designed RCTs that combine the different aspects of the lifestyle and are personalized to achieve better clinical and cost effectiveness.

## Background

GDM is defined as the presence of hyperglycaemia, first detected any time during pregnancy [1]. Globally around, 20 million or 16% of all live births are affected by hyperglycaemia during pregnancy, of which more than 90% are present in LMICs [2]. Typically, GDM is diagnosed between 24 and 28 weeks of gestation [3]. However, varying degrees of hyperglycaemia may be present before this time period [4] and adverse effects on the fetus may have already happened at the time of diagnosis of GDM [5–7].

Maternal metabolic health during pregnancy has critical influence on the metabolic health of the offspring and possibly even in the subsequent generations [8]. There is increasing evidence that pre-conception health of women is of critical importance in shaping the metabolic health of the next generation [9–11]. In addition, GDM has been shown to be associated with adverse fetal programming [12]. Even in research setting, current management strategy for GDM, at best, reduces the short-term complications by about 50% [13, 14]. Thus, the focus needs to move away from “diagnosis and treatment of GDM” to “prevention of GDM.” Prevention of GDM may provide a crucial opportunity to reduce this risk of adverse metabolic programming of the offspring and future risk of cardiometabolic disorders, in addition to benefiting the mothers [15].

Lifestyle interventions are proven to effectively prevent type-2 diabetes [16]. Hence it is conceivable that such lifestyle interventions could prevent GDM. However, studies that tested the effectiveness of lifestyle interventions provided during pregnancy on GDM have shown mixed results [14, 17–21]. Interestingly, interventions provided during early weeks of gestation showed promising results [22]. Data from International Weight Management in Pregnancy Collaborative (iWIP) suggest that with careful selection of subjects and the type of intervention, GDM can be prevented if the interventions are carried out in early pregnancy [23]. A recent meta-analysis shows that antenatal structured diet and physical activity-based lifestyle interventions were linked to reduced gestational weight gain and lower risk of other adverse maternal and neonatal outcomes [23] and two individual participant data (IPD) meta-analysis on prevention of GDM are ongoing [24, 25]. However, this approach may still not reduce the adverse programming that may happen at or soon after the time of conception. Therefore, the best way to abolish this excess risk is by prevention of GDM with interventions before pregnancy.

Two recent studies have shown that lifestyle interventions before pregnancy may help to prevent GDM. However, these studies are small, of varying quality and used different components of “lifestyle interventions” [26, 27]. Not many have reported in LMICs, where the biggest burden of GDM is present. This systematic review aimed to summarize the available evidence from randomized control trials (RCTs), case-control studies and cohort studies for the various components of pre-pregnancy lifestyle in reducing the risk of GDM. Where possible, we conducted meta-analysis of the studies to quantify the effect of these components.

## Methods

### Search strategy

This is a systematic review and meta-analysis summarizing the evidence linking “pre-pregnancy” lifestyle (diet and/or physical activity aspects) and GDM risk and reporting the results of the analysis using a robust estimate, that is, Odds Ratio (OR). The PI(E)CO framework for this study is as follows: Population: Pregnant women, Intervention/Exposure: Pre-pregnancy diet and/or physical activity-based lifestyle intervention/exposure, Comparison: No pre-pregnancy diet and/or physical activity-based lifestyle intervention/exposure, Outcomes: Prevention of GDM/reduced risk of developing GDM.

The following electronic databases were searched: MEDLINE, Web of Science, Embase and Cochrane Central Register of Controlled Trials (CENTRAL). The search term combinations were designed with appropriate MeSH, free text and word variants to capture all studies on “Pre-pregnancy lifestyle interventions/components preventing Gestational Diabetes Mellitus.”

The search carried out were “[(Pregnant women OR Pregnancy OR Pre-pregnancy) AND (Lifestyle intervention OR Diet OR Exercise OR Behavioral change intervention OR Lifestyle education) AND (Gestational Diabetes OR Gestational Diabetes Mellitus OR Hyperglycaemia during Pregnancy)]”. All studies from inception till July 2022 were searched. All database searches were limited to “human” studies in order to eliminate animal model and other irrelevant studies. No Language restrictions were applied. The protocol was registered in PROSPERO (ID: CRD42020189574).

### Study selection

#### Study inclusion

Studies that are randomized control trials (RCTs), cohort studies and case-control studies that assessed the effect of pre-pregnancy lifestyle (Diet and/or physical activity based) in preventing GDM were included.

#### Study exclusion

Studies involving women with type-1 or type-2 diabetes, women aged <18 and more than 50 years, women on metformin therapy (up to 6 weeks before) for anovulation and/or infertility, women with severe anaemia defined as hemoglobin (Hb) < 80 g/L, sickle cell traits, sickle cell anaemia and other genetic Hb variants and any other serious medical illness, any lifestyle intervention started only during pregnancy, drug interventions unless they had a separate lifestyle arm to prevent GDM, lifestyle intervention that have not reported incidence of GDM in their outcomes were excluded.

Two reviewers (SS and DP) independently performed the study selection. Rayyan, a web-based tool, was used to do the screening and selection. Any conflicts arising in study selection was resolved after a discussion. A third reviewer (NS) resolved any conflict arising in study selection or during quality assessment. Reference lists and gray literature were also searched to capture any unpublished data and additional studies. Following initial title and abstract screening, full-text screening was carried out.

### Data extraction

Two independent authors extracted data of the selected studies. The data extracted were author, year, type of lifestyle intervention, time of lifestyle intervention/healthy lifestyle exposure, number of participants, ethnicity, country of study, mean BMI, mean age, Odds Ratio, Relative risk and other reported results. We extracted data on the number of events (GDM and non-GDM) in women exposed and not exposed to any lifestyle factors.

### Risk of bias assessment

Two independent reviewers assessed the quality of the selected studies. All the following sources of bias were addressed: blinding, sequence generation, incomplete outcome data, allocation concealment, selective outcome reporting and others. The Cochrane ROB (Risk of Bias)-2 tool was used for assessing the quality of the selected randomized control trials. The Newcastle-Ottawa Scale (NOS) for assessing the quality of non-randomized studies. Based on the NOS, (0–9 for case-control studies / 0–10 for cohort studies) studies were categorized as follows: low risk (6–9/10), some concerns (3–5), high risk (0–2). The Publication bias and small studies effect quality assessment was carried out by assessing funnel plots using Egger’s tests.

### Strategy for data synthesis

The outcome assessed is the risk of GDM. Random effects model using Mantel-Haenszel method was used to calculate odds ratio (OR) with 95% confidence interval from the relevant summary estimates reported by the included studies. Studies from the same study cohort are considered only once for meta-analysis but are listed in the summary table if any useful insight are provided based on components of intervention/exposure. For cohort studies that reported the events of GDM and non-GDM for various categories of diet and/or physical activity measures, studies with similar exposure group (low carb intake/low sugar diet group, higher quality diet scores, high physical activity group) were taken into consideration for meta-analysis. Cohort studies that report association of GDM and different components of diet and physical activity are included as separate studies for the purpose of this review. Statistical heterogeneity between the studies was assessed using I^2^ statistics. RevMan software version 5.4.1 was used for the statistical analysis.

### Role of the funding source

The funder of the study had no role in study design, data collection, data analysis, data interpretation, or writing of the report.

## Results

**Fig. 1:**
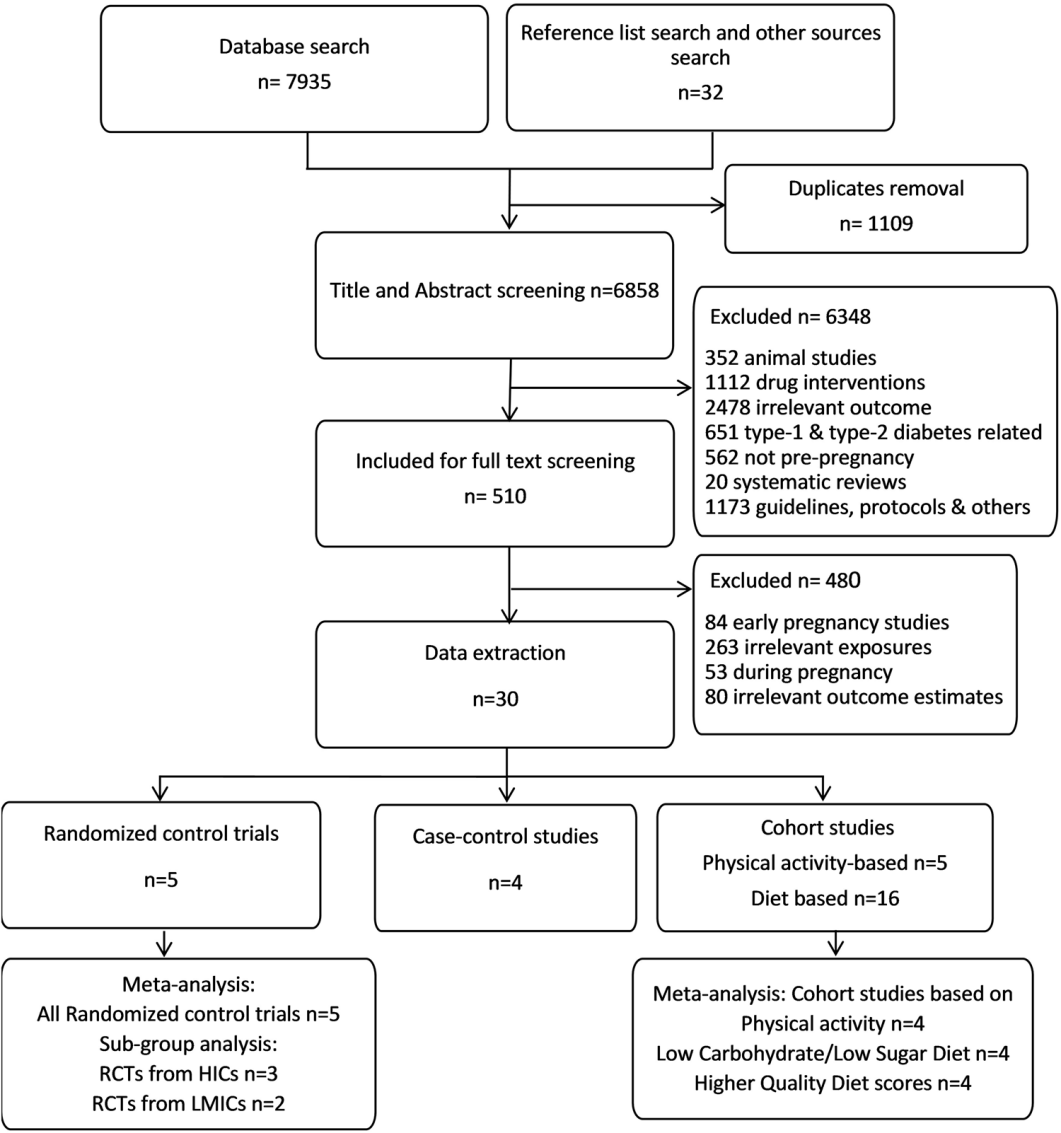
Study selection. Number of studies selected as per the inclusion and exclusion criteria; PRISMA flow chart.

The initial databases search found 7935 studies and an additional 32 studies were identified from other sources (Fig. 1). Removing duplicates and abstract screening resulted in 510 studies for full-text screening and 30 were tabulated (Tables 1–4). Five of them were RCTs (*n* = 2471 pregnancies) [28–32], 4 case-control studies (*n* = 19,778 pregnancies) [33–36], and 21 cohort studies (*n* = 235,627 pregnancies). Of these, 5 were physical activity based (*n* = 46197 pregnancies) [27, 37–40], and 16 were diet based exploring various components (*n* = 189,430 pregnancies) [27, 41–55]. Full text of one of the RCTs was in Russian and hence only the data from the abstract was used [28]. The study characteristics based on the type and lifestyle components were summarized in Tables 1–4. Three RCTs tested a combination of diet and physical activity factors from high income countries. Two RCTs tested diet and were from low- and middle-income countries.

**Table 1:** Study characteristics of the RCTs (*n* = 5).

Author, Year	Study name, duration	Participant size(n), Country, mean(SD) BMI kg/m2, mean(SD) age years	Type of Intervention, time of intervention	Intervention assessment	GDM assessment	Results reported: Odds Ratio/Relative risk (95%CI, p- value)/GDM incidence
Makarova et al., 2020 [28]	-, Pre-pregnancy and course of pregnancy	*n* = 98, Russia, I = 36, C = 62	Pregravid preparation, Pre-pregnancy	Comprehensive pregnancy preparation program (diet therapy, replenishment of macro- and micronutrients: vitamin D3, folic acid, iodine, iron, and an anti-obesity drug combination of sibutramine + metformin)	Russian national consensus 2012	GDM developed in 50% women of the comparison group and in 11.1% women of the intervention group
Rono et al., 2018 [29]	The Finnish RADIEL study, between the years 2008 and 2014	*n* = 128, I = 65, C = 63, Finland, BMI: I = 30.5 (6.3) C = 28.1 (5.7) Age: I = 32 (5) C = 32 (4)	Individually modifiable dietary and physical activity counseling from trained study nurses, Pre-pregnancy (1 year)	Trained study nurses provided individualized lifestyle counseling every 3 months in addition to a group session with a dietician. The control group received standard antenatal care	GDM was defined as one or more pathological glucose values in a 75 g 2-hour oral glucose tolerance test, performed between 12 and 16 weeks of gestation and if normal repeated between 24 and 28 weeks of gestation	Cumulative incidence: I = 60% (*n* = 39/65), C = 54% (*n* = 34/63) *p* = 0.49
Valkama et al., 2018 [32]	-, The duration of treatment is from the time of enrollment to the 20-week gestation period	*n* = 75, Finland, BMI: I = 30.4 (6.1) C = 27.9 (5.8) Age: I = 32.4 (4.1) C = 31.5 (3.9)	Lifestyle counseling on food intakes, Pre-pregnancy	Food intakes were followed from pre-pregnancy to early pregnancy using a food frequency questionnaire	A 75-g oral glucose tolerance test conducted in the first and second trimester of pregnancy	GDM incidence was 45% (*n* = 17) in the control group and 59% (*n* = 22) in the intervention group (*p* = 0.20)
Sahariah et al., 2016 [30]	Project SARAS, (2006–2012)	*n* = 1008, I = 492, C = 516 India, BMI: I = 19.6 (17.7, 22.3) C = 20.1 (17.9, 22.8) Age: I = 24.0 (21.0, 27.0) C = 25.0 (22.0, 27.0	A daily snack containing leafy green vegetables, fruit, and milk, Pre-pregnancy till pregnancy	The interventions included a daily snack made from leafy green vegetables, fruit, and milk for the treatment group or low-micronutrient vegetables (e.g., potato and onion) for the control group, in addition to the usual diet	WHO 1999 criteria	OR: 0.56 (0.36–0.86, 0.008)
Sun et al., 2020 [31]	-, February 2016 to October 2018	*n* = 1162, I = 582, *P* = 580, China, BMI: I = 22.47 ± 3.66 C = 22.61 ± 4.01 Age: I = 31.2 ± 4.5 C = 31.4 ± 4.3	100 g mushroom daily, Pre‐pregnancy to the 20th week of gestation	Subjects were required to consume 100 g mushroom daily from pre-pregnancy to the 20th week of gestation	IADPSG criteria	Normal weight : OR 2.66 (1.38–3.62, 0.024) Over weight : OR 2.11 (1.09–4.43, 0.082)

*I* Intervention group, *C* Control group, *SD* Standard Deviation, *CI* Confidence interval, *OR* Odds Ratio, *RR* Relative Risk, *IADPSG* International Association of Diabetes and Pregnancy Study Groups, *WHO* World Health Organization.

**Table 2:** Study characteristics of the case-control studies (*n* = 4).

Author, Year	Study duration	Participant size, country, mean(SD) BMI kg/m^2^, mean(SD) age years	Type of Exposure, Time of Exposure	Intervention assessment	GDM assessment	Results reported: Odds Ratio or Relative risk (95%CI, *p* value) or GDM incidence
Asadi et al., 2019 [33]	September 2014 and March 2015	*n* = 278, Iran, Pre-pregnancy weight: (kg) GDM:68.82 ± 13.31 Control:63.02 ± 11.77 Age: GDM:29.00 ± 5.17 Control:27.50 ± 4.92	Dietary patterns (Prudent diet-higher intakes of fruits, low-fat dairy, potato, Egg, fish, poultry, nuts, organs meat and red meat), Pre-pregnancy	Dietary assessment was carried out by using a 67-item validated food frequency questionnaire to evaluate dietary history of participants during the last year	ADA criteria	Prudent Diet: OR 0.88 (0.44–0.99, 0.01) Western Diet: OR 1.50 (0.74–3.03, 0.2)
Chen et al., 2019 [35]	March 1, 2012, and December 30, 2016	*n* = 9556, China	Dietary nutrient patterns (High vitamin intake), One year before conception	A “vitamin” nutrient pattern was characterized as the consumption of diet rich in vitamin A, carotene, vitamin B2, vitamin B6, vitamin C, dietary fiber, folate, calcium, and potassium.	IADPSG criteria	For every quartile increase in the vitamin factor score during one year prior to conception, the GDM risk decreased by 9%; OR: 0.91 (0.86–0.96)
Chen et al., 2020 [34]	March 1, 2012 and December 30, 2016	*n* = 9556, China	Dietary nutrient patterns (High vegetable intake), One year before conception	A “vegetable” dietary pattern was characterized as the consumption of green leafy vegetables (Chinese little greens and bean seedling), other vegetables (cabbages, carrots, tomatoes, eggplants, potatoes, mushrooms, peppers, bamboo shoots, agarics, and garlic), and bean products (soybean milk, tofu, kidney beans, and cowpea)	IADPSG criteria	For every quartile increase in the vegetables factor score during 1 year prior to conception, the GDM risk lowered by 6%; OR: 0.94 (0.89 to 0.99)
Shivappa et al., 2019 [36]	-	*n* = 388, Iran, BMI: 27.25 (3.82) (GDM) vs 24.64 (3.32) (non-GDM) Age: 29.64 (4.52) (GDM) vs 29.76 (4.26) (non-GDM)	Dietary inflammatory index (DII) scores using 147-item food frequency questionnaire (Low DII), 1 year before the dietary assessment at 24–28 weeks of gestation	DII scores (energy, carbohydrate, protein, total fat, fiber, cholesterol, saturated fat, mono-unsaturated fat, poly unsaturated fat, omega-3, omega-6, trans fat, niacin, thiamin, riboflavin, vitamin B12, vitamin B6, iron, magnesium, selenium, zinc, vitamin A, vitamin C, vitamin D, vitamin E, folic acid, beta carotene, garlic, turmeric, onion, caffeine)	Carpenter and Coustan criteria	Continuous DII: OR 1.2 (0.94–1.54) Categorical DII Tertile 3 vs 1: OR: 2.1 (1.02–4.34)

*SD* Standard Deviation, *CI* Confidence interval, *OR* Odds Ratio, *RR* Relative Risk, *IADPSG* International Association of Diabetes and Pregnancy Study Groups, *ADA* American Diabetes Association.

**Table 3:** Study characteristics of other cohort studies (Physical activity- PA based *n* = 5).

Author, Year	Study name, duration	Participant size, country, mean (SD) BMI kg/m^2^, mean (SD) age years	Type of Exposure, time of exposure	Intervention assessment	GDM assessment	Results reported: Odds Ratio or Relative risk (95%CI, *p* value) or GDM incidence
Currie et al., 2014 [37]	-, October 2002 to July 2005	*n* = 1,749, Canada, Mean age was 31 years, 49 % were nulliparous, and 41 % had a prepregnancy BMI of 25 kg/m2 or higher	Physical activity (High), a year before pregnancy	The Kaiser Physical Activity Survey, completed at approximately 20 weeks’ gestation, requested information regarding physical activity during the year before the pregnancy and the first 20 weeks of pregnancy. Outcomes were assessed by medical chart review	SOGC guidelines	Associations were not observed between total physical activity and gestational diabetes
Dempsey et al., 2004 [38]	OMEGA Study, 1996–2000	*n* = 909, USA	Recreational physical activity (High), a year before pregnancy	Women were questioned during early gestation about physical activity performed during the year before and 7 days prior to the interview during pregnancy	National Diabetes Data Group criteria	RR: 0.24 (0.10, 0.64)
Oken et al., 2006 [39]	Project Viva, 1999 to 2002	*n* = 1,805 USA, Mean (standard deviation [SD]) age was 32.1 (5.0) years, and prepregnancy BMI was 24.6 (5.2) kg/m2	Physical activity (High), a year before pregnancy	Assessed duration and intensity of physical activity and time spent viewing television both before and during pregnancy	National Diabetes Data Group criteria	OR: 0.56 (0.33–0.95)
Zhang et al., 2006 [40]	Nurses’ Health Study II, Between 1990 and 1998	*n* = 21,765, USA	Amount and intensity of pre-gravid physical activity and sedentary behavior (High), Pre-pregnancy	Physical activity and sedentary behaviors were assessed through mailed questionnaires	National Diabetes Data Group criteria	RR: 0.77 (0.69–0.94, 0.002)
Zhang et al, 2014 [27]	Nurses’ Health Study II, 1989–2001	*n* = 14437, USA	Combination of lifestyle factors (Smoking, diet and physical activity) (High), Pre-pregnancy	Questionnaires are administered biennially to update lifestyle characteristics	National Diabetes Data Group criteria	RR: 0.59 (0.48–0.71)

*SD* Standard Deviation, *CI* Confidence interval, *OR* Odds Ratio, *RR* Relative Risk, *SOGC* The Society of Obstetricians and Gynaecologists of Canada.

**Table 4:** Study characteristics of other cohort studies (Diet based *n* = 16).

Author, Year	Study name, duration	Participant size, country	Type of Exposure, time of Exposure	Intervention assessment	GDM assessment	Results reported: Odds Ratio or Relative risk (95%CI, *p* value) or GDM incidence
Bao et al., 2015 [42]	Nurses’ Health Study II (NHS II), 1989–2001	*n* = 21,411, USA	Low carbohydrate diet scores (A higher score indicates higher intake of fat and protein and lower intake of carbohydrate), Pre-pregnancy	Prepregnancy LCD scores were calculated from validated food-frequency questionnaires	National Diabetes Data Group criteria	Overall LCD score RR: 1.27 (1.06–1.51, 0.03)
Chen et al., 2009 [45]	Nurses’ Health Study II, 1989–2001	*n* = 13,475, USA	Low intake (0–3 servings/month) of sugar-sweetened beverage consumption vs high intake (1–4 servings/week and >5 servings/week), Pre-pregnancy	Dietary intake information was collected by a 133-item SFFQ designed to assess average food intake over the previous year. For beverages, one serving (considered as one glass, bottle, or can) was used as the unit for the consumption of ssbs	National Diabetes Data Group criteria	RR:1.22 (1.01–1.47, 0.04)
Looman et al., 2018 [47]	Australian Longitudinal Study on Women’s Health (ALSWH), 2003 and 2015	*n* = 3607, Australia	Dietary carbohydrate quantity and quality (Low carb & high fiber), Pre-pregnancy	Diet was assessed using the Dietary Questionnaire for Epidemiological Studies (DQES)	Diagnostic criteria for GDM in Australia	Low carb & High fiber RR: 0·67 (0·45, 0·96)
Mikel et al., 2018 [50]	SUN cohort, December 1999 and March 2012	*n* = 3396, Spain	Low Soft drink consumption (Low risk - rarely or never or <1/month), Pre-pregnancy	A validated 136-item semi-quantitative food frequency questionnaire was used to assess soft drink consumption	National Diabetes Data Group criteria	High consumption: OR 2.03 (1.25–3.31)
Lynn et al., 2020 [48]	He Nulliparous Pregnancy Outcomes Study: Monitoring Mothers-To-Be (numom2b), 2010 to 2013	*n* = 8259, USA	High Healthy Eating Index (HEI) – 2010 score, Pre-pregnancy (3 months around conception)	Women completed the modified Block 2005 Food Frequency Questionnaire, a semiquantitative assessment of usual dietary intake for the 3 months around conception	National Diabetes Data Group criteria	There were no differences in frequency of GDM or cesarean delivery by HEI quartile on bivariable analysis
Mikel et al., 2019 [51]	SUN cohort, December 1999 and March 2012	*n* = 3455, Spain	High DDS Dietary-Based Diabetes- Risk Score, Pre-pregnancy	A validated 136-item semi-quantitative FFQ was used to assess pre-gestational dietary habits	National Diabetes Data Group criteria	OR: 0·48 (0·24, 0·99, 0·01)
Tobias et al., 2012 [53]	Nurses’ Health Study II, 1989–2001	*n* = 21, 376, USA	High Alternative Healthy eating index scores (derived from USDA Food Guide Pyramid and the 1995 Dietary Guidelines for Americans), Pre-pregnancy	Prepregnancy dietary pattern adherence scores were computed based on participants’ usual intake of the patterns’ components, assessed with a validated food-frequency questionnaire	National Diabetes Data Group criteria	HEI RR: 0.54 (0.43–0.68, <0.0001)
Zhang et al., 2014 [27]	Nurses’ Health Study II, 1989–2001	*n* = 13,110, USA	High Alternate Healthy Eating Index-2010 diet score, Pre-pregnancy	Every four years thereafter, participants were asked to complete a semiquantitative food frequency questionnaire in addition to the main questionnaire	National Diabetes Data Group criteria	Total fiber: RR 0.70 (0.52–0.96)
Amelia et al., 2017 [41]	SUN cohort, December 1999 and March 2012	*n* = 3298, Spain	Low Meat consumption and iron intake (Median 33.7 g/day), Pre-pregnancy	Meat consumption and iron intake were assessed at baseline through a validated, 136-item semi-quantitative, food frequency questionnaire.	National Diabetes Data Group criteria	Total meat: OR 1.67 (1.06–2.63, 0.010)
Bao et al., 2014 [42]	Nurses’ Health Study II (NHS II), 1989–2001	*n* = 15027, USA	Fried food consumption (Low), Pre-pregnancy	Collected diet information, including consumption of fried foods at home and away from home, using a validated food frequency questionnaire	National Diabetes Data Group criteria	For who consumed Fried food >7 times/week RR: 2.18 (1.53–3.09, <0.001)
Bao et al., 2018 [43]	Nurses’ Health Study II (NHS II), 1989–2001	*n* = 15225, USA	Habitual intake of vitamin D from diet and supplements (High vitamin), Pre-pregnancy	Diet information, including vitamin D intake from food sources and supplements, was assessed by validated food frequency questionnaires	National Diabetes Data Group criteria	RR 1–399 IU/day: 0.80 (0.67–0.96, 0.002) ≥400 IU/day: 0.71 (0.56–0.90, 0.002)
Li et al., 2019 [46]	Nurses’ Health Study II, 1989–2001	*n* = 14,553, USA	Adequate Habitual Intakes Folate (from food and supplement) ( ≥400 μg/day), Pre-pregnancy	Prepregnancy intakes of total folate, supplemental folate, and food folate were assessed using a food frequency questionnaire administered every 4 years	National Diabetes Data Group criteria	OR: 0.83 (0.72, 0,95, 0.007)
Mikel et al., 2017 [49]	SUN cohort, December 1999 and March 2012	*n* = 3455, Spain	Adherence to Mediterranean dietary pattern, Pre-pregnancy	The 136 food items included in the FFQ were classified in twenty-six predefined food groups.	National Diabetes Data Group criteria	OR: 0·24 (0·10, 0·55)
Schoenaker et al., 2015 [52]	Australian Longitudinal Study on Women’s Health (ALSWH), 2003 and 2015	*n* = 3853, Australia	Low risk diet- low meats, snacks and sweets consumption, Pre-pregnancy	Pre-pregnancy dietary patterns were derived using factor analysis based on 101 food items from a validated food frequency questionnaire	Diagnostic criteria for GDM in Australia	OR: 0.85 (0.76, 0.98)
Zhang et al., 2006 [54]	Nurses’ Health Study II, 1989–2001	*n* = 13,110, USA	Low risk prudent diet (positively correlated with intakes of fruits, green leafy vegetables, poultry and fish), Pre-pregnancy	Dietary intake information was collected using a 133-food item semi-quantitative FFQ designed to assess average food intake over the previous year	National Diabetes Data Group criteria	Prudent pattern RR: 1.39 (1.08–1.80, 0.018)
Zhang et al., 2006 [55]	Nurses’ Health Study II, 1989–2001	*n* = 14,437, USA	High fiber intake ( >22 g/day), Pre-pregnancy	Dietary intake information was collected by a 133-food SFFQ designed to assess average food intake over the previous year	National Diabetes Data Group criteria	RR: 0.81 (0.70–0.94)

*SD* Standard Deviation, *CI* Confidence interval, *OR* Odds Ratio, *RR* Relative Risk.

**Fig. 2:**
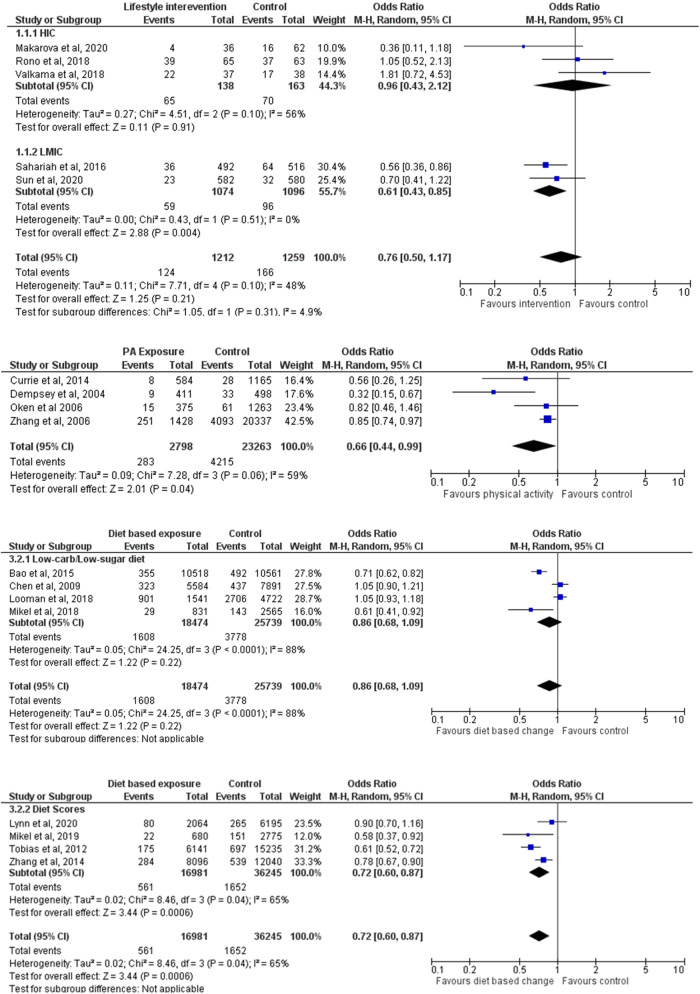
Meta-analysis of pre-pregnancy lifestyle intervention and risk of GDM. The 4 panels show the different components of lifestyle interventions and risk of GDM.

Meta-analysis were carried out to assess the pooled effect size by study type and components of lifestyle measures. Figure 2a shows the effect of pre-pregnancy RCTs of lifestyle intervention. 3 out of 5 of these studies were dietary interventions. Women in the intervention group were 24% less likely to develop GDM but this was not statistically significant (OR 0.76, 95% CI: 0.50–1.17, *p* = 0.21). Sub-group analysis of the studies from HICs and LMICs was also performed as shown in Fig. 2a. These studies had moderate heterogeneity. Quality assessment of these RCTs revealed that only 20% of the studies had low risk of bias. The bias was mainly due to randomization process and missing outcome data (Supplementary Fig. 1).

Three of the case-control studies studied dietary patterns and other case-control study focused on Dietary Inflammatory Index (DII). Meta-analysis of case-control studies was not performed as 2 studies are from the same study population and the dietary exposures are very heterogenous to combine in a meta-analysis. Quality assessment of the case-control studies revealed that low risk of bias with Newcastle-Ottawa scale score 8 (Supplementary Table 1)

**Fig. 3:**
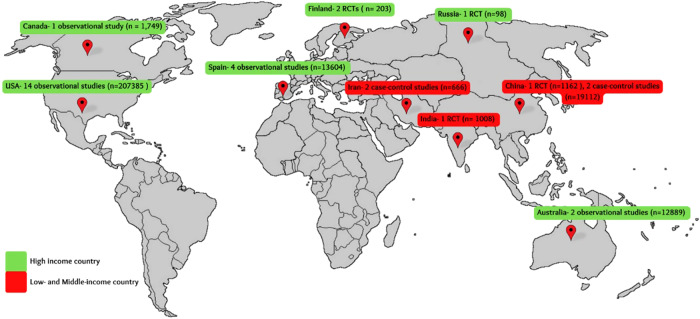
Distribution of studies across the world. Worldwide distribution of included studies linking pre-pregnancy lifestyle and GDM risk.

Meta-analysis of cohort studies (*n* = 4) revealed that women who were more physically active before pregnancy were 34% less likely to develop GDM (OR 0.66, 95% CI: 0.44, 0.99, *p* = 0.04) (Fig. 2b). Similarly, women who had low carbohydrate/low sugar diet were 24% less likely to develop GDM (OR 0.86, 95% CI 0.68–1.09, *p* = 0.22) (Fig. 2c). Meta-analysis (Fig. 2d) of cohort studies linking diet score and GDM showed that women with higher quality diet scores were 28% less likely to develop GDM (OR 0.72, 95%CI: 0.60, 0.87, *p* = 0.0006). These studies had substantial heterogeneity among them. Quality assessment of the cohort studies revealed low risk of bias with Newcastle-Ottawa scale score 8 (Supplementary Table 1). 24 of all 30 studies were from high income countries and 6 were from low- and middle- income countries (Fig. 3). Supplementary Figs 2a-2d show the funnel plots for publication bias, which reveal symmetry and show very low risk of publications bias. Supplementary Figs 3a-3d show the sensitivity analysis based on leave-one out plots confirming the robustness of the results reported.

## Discussion

Our systematic review and meta-analysis summarize crucial evidence highlighting the importance of “pre-pregnancy” lifestyle in reducing the risk of developing GDM. To our knowledge, our systematic review and meta-analysis is the first to show the evidence for pre-pregnancy lifestyle interventions and the risk of GDM. Data from RCTs were limited and showed that pre-pregnancy lifestyle interventions may reduce the risk of developing GDM by about 24%. The cohort studies explored various components of diet and physical activity measures and confirmed a strong link between higher self-reported physical activity and diets that are classified as “healthy” during the pre-pregnancy period and reduced risk of GDM. Analysis of cohort studies on low carbohydrate/low sugar diet and higher quality diet scores also showed positive effect in reducing the risk of developing GDM.

A recent systematic review during pregnancy showed that structured dietary intervention can reduce the risk of GDM by about 39% in 3029 women [23] The findings from our RCTs however did not show a significant reduction in the risk of GDM in women who received lifestyle intervention before pregnancy. This may be due to the heterogeneity of the studies using different components of the lifestyle intervention and due to the smaller number of pre-pregnancy trials conducted thus far. Nevertheless, it provides crucial evidence that lifestyle intervention before pregnancy is doable. Additionally, well-designed RCTs are required to assess the benefits of different components of dietary intervention. Interventions that focussed on physical activity during early pregnancy have been shown to reduce the development of GDM [23, 27] and type 2 diabetes in at risk individuals [16, 56]. While we did not find any RCTs that focussed only on physical activity alone interventions before pregnancy, the cohort studies summarize the available evidence and highlight the need for RCTs.

### Strengths and limitations

Our study used an extensive search strategy and a robust methodology to include and evaluate the different components of dietary and physical activity interventions in the pre-pregnancy period. Inclusion of non-RCTs studies, provided a comprehensive summary and highlighted the need for future RCTs on different components of lifestyle interventions before pregnancy to reduce the risk of GDM. It also highlights the paucity of evidence from LMICs where the burden of GDM is very high. Our study however has the following limitations. Firstly, there is high heterogeneity between the studies. This is likely be due to the various different lifestyle factors that are included in these studies. Secondly, most of these cohort and case-control studies were based on food frequency and physical activity questionnaires, which would result in self-reported bias. This would need to be kept in mind and future studies should ideally use objective measures to minimize this bias. Finally, not many studies were conducted from LMICs and therefore our findings were not applicable to populations who have the highest risk of GDM.

### Implications for future research

Our review also highlights the paucity of data from LMICs, which may highlight the potential difficulties of conducting RCTs, especially in the pre-pregnancy period. However, high birth rate in LMICs provide other windows of opportunities such as “inter-pregnancy” interval [26]. This may also provide opportunities for targeting women at high-risk, for example those who had GDM in their previous pregnancy, which may be more cost effective.

While our review highlights the potential benefits of various components of diet and lifestyle measures, studies that focus on reducing sedentary behavior are needed. Sedentary behavior is associated with higher metabolic and cardiovascular risk [57, 58] and break in sedentary behavior has been shown to improve glucose and metabolic profile in women post-menopause [59]. This approach may work during inter-pregnancy interventions, especially in women with previous history of GDM with young children.

Finally, future interventions should be co-developed and “personalized.” It is known that women from high-risk ethnic groups have difficulty in following “generalized” lifestyle intervention [60, 61]. Thus, it is critically important that these women receive a “personalized” lifestyle intervention that relates closely to their lifestyle. Newer technologies such as mobile phone based remote interventions offer promise that these can be achieved at a lower cost [62, 63]. Face-to-face physical activity interventions can be challenging in women with young children [64] and may increase the travel cost and time burden in LMICs. In LMICs, mobile phone connections are increasing and more accessible than clean water [65]. Thus, using mobile phone technology to deliver these interventions would be pragmatic and urgently needed.

### Supplementary information

SUPPLEMENTAL MATERIAL

## Data Availability

The data extracted and analyzed in this systematic review and meta-analysis are available from the corresponding author upon request.
