# Supplementary material for: Effectiveness of pre-pregnancy lifestyle in preventing gestational diabetes mellitus—a systematic review and meta-analysis of 257,876 pregnancies
Source: Nutr Diabetes. 2023 Nov 16;13:22. doi: 10.1038/s41387-023-00251-5 (PMC10654718; doi:10.1038/s41387-023-00251-5)
Supplement: Supplementary file 1 — SUPPLEMENTAL MATERIAL [file 41387_2023_251_MOESM1_ESM.docx]

**
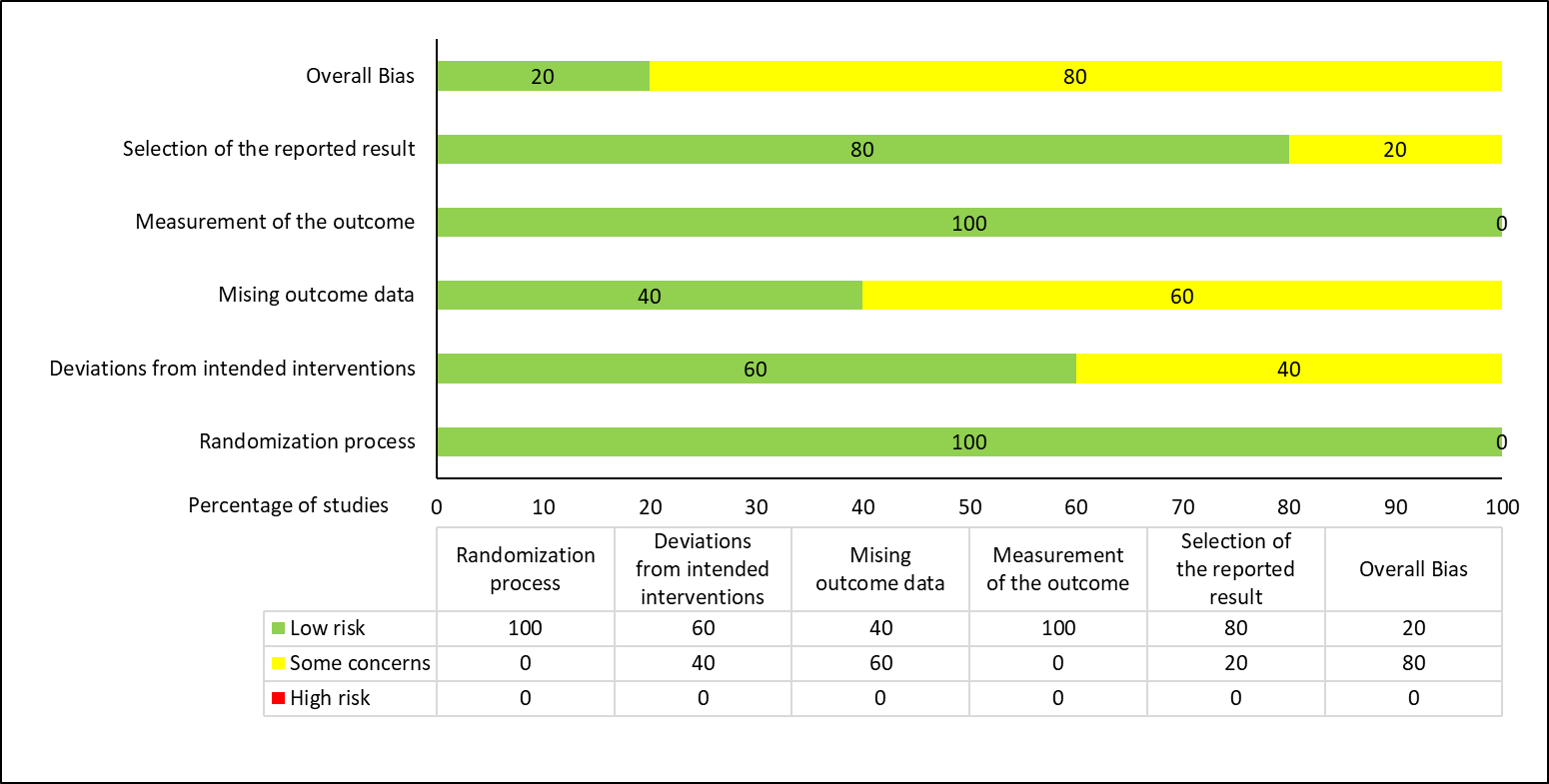
Supplementary Figure 1: Risk of bias assessment for RCTs using Cochrane’s Risk of Bias (ROB) tool -2**

**Supplementary Table 1: Risk of bias assessment for case-control and cohort studies using Newcastle-ottawa scale of star scoring: low risk (6-9/10), some concerns (3-5), high risk (0-2)**

| **Case control studies** | **Selection** | | | | **Comparability** | **Exposure** | | |
| --- | --- | --- | --- | --- | --- | --- | --- | --- |
|  | **1*** | **2*** | **3*** | **4*** | **1**** | **1*** | **2*** | **3*** |
| Asadi et al 2019 | * | * | * | * | ** |  | * | * |
| Chen et al 2019 | * | * | * | * | ** |  | * | * |
| Chen et al 2020 | * | * | * | * | ** |  | * | * |
| Shivappa et al 2019 | * | * | * | * | ** |  | * | * |
| **Cohort studies** | **Selection** | | | | **Comparability** | **Outcome** | | |
| **Cohort (PA based) studies** | **1*** | **2*** | **3*** | **4*** | **1**** | **1*** | **2*** | **3**** |
| Currie et al 2014 | * | * |  | * | ** | * | * | * |
| Dempsey et al 2004 | * | * |  | * | ** | * | * | * |
| Oken et al 2006 | * | * |  | * | ** | * | * | * |
| Zhang et al 2006 | * | * |  | * | ** | * | * | * |
| Zhang et al 2014 | * | * |  | * | ** | * | * | * |
| **Cohort (Diet based) studies** | **1*** | **2*** | **3*** | **4*** | **1**** | **1*** | **2*** | **3*** |
| Amelia et al 2017B | * | * |  | * | ** | * | * | * |
| Bao et al 2014 | * | * |  | * | ** | * | * | * |
| Bao et al 2015 | * | * |  | * | ** | * | * | * |
| Bao et al 2018 | * | * |  | * | ** | * | * | * |
| Chen et al 2009 | * | * |  | * | ** | * | * | * |
| Li et al 2019 | * | * |  | * | ** | * | * | * |
| Looman et al 2018 | * | * |  | * | ** | * | * | * |
| Lynn et al 2020 | * | * |  | * | ** | * | * | * |
| Mikel et al 2017 | * | * |  | * | ** | * | * | * |
| Mikel et al 2018 | * | * |  | * | ** | * | * | * |
| Mikel et al 2019 | * | * |  | * | ** | * | * | * |
| Schoenaker et al 2015 | * | * |  | * | ** | * | * | * |
| Tobias et al 2012 | * | * |  | * | ** | * | * | * |
| Zhang et al 2014 | * | * |  | * | ** | * | * | * |
| Zhang et al (2), 2006 | * | * |  | * | ** | * | * | * |
| Zhang et al (3), 2006 | * | * |  | * | ** | * | * | * |

**Supplementary Figure 2: Funnel plots for**

1. **RCTs (n=5)**

**
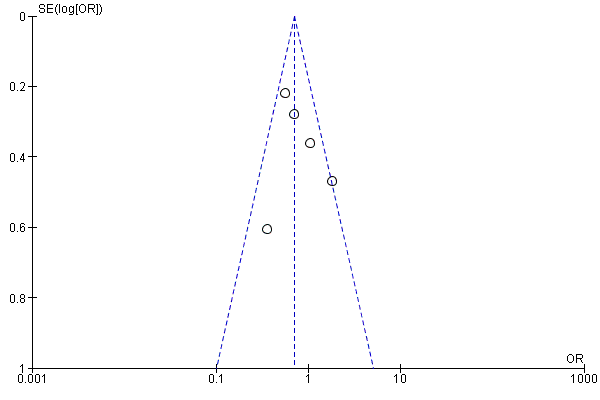
**

1. **Case-control studies (n=4)**

**
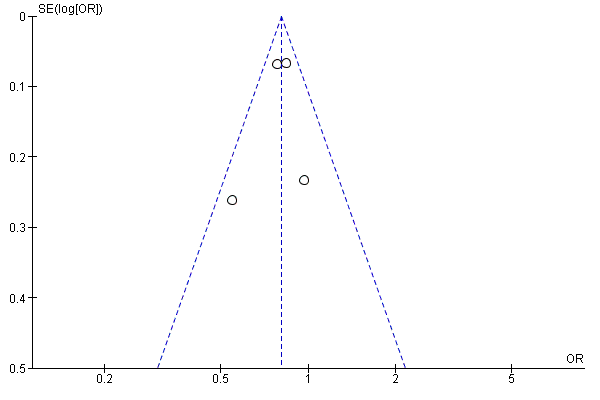
**

1. **Cohort studies based on Physical activity (n=5)**

**
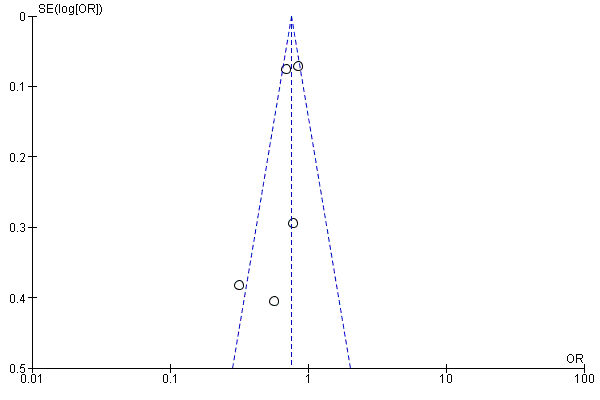
**

1. **
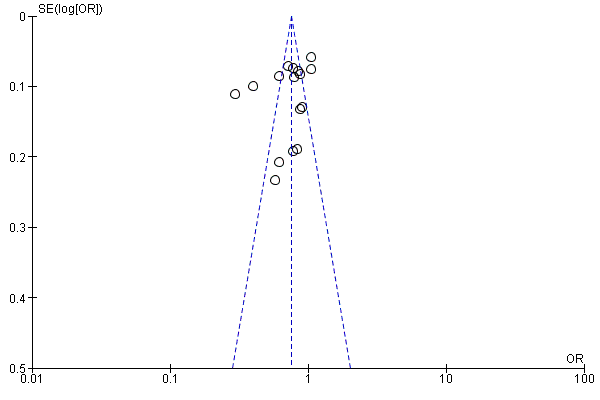
Cohort studies based on Diet (n=16)**

**
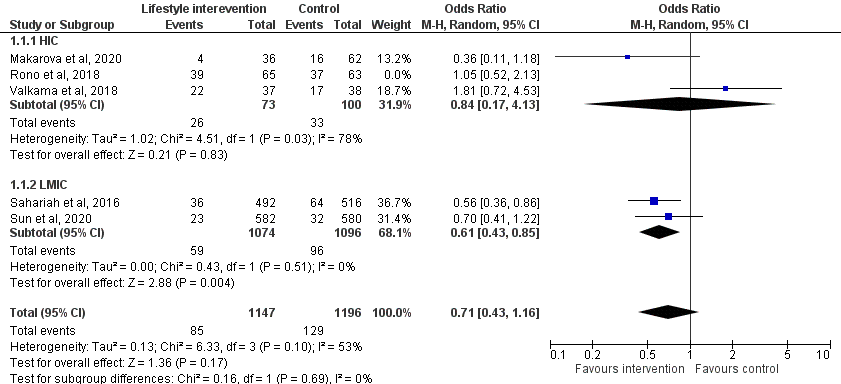

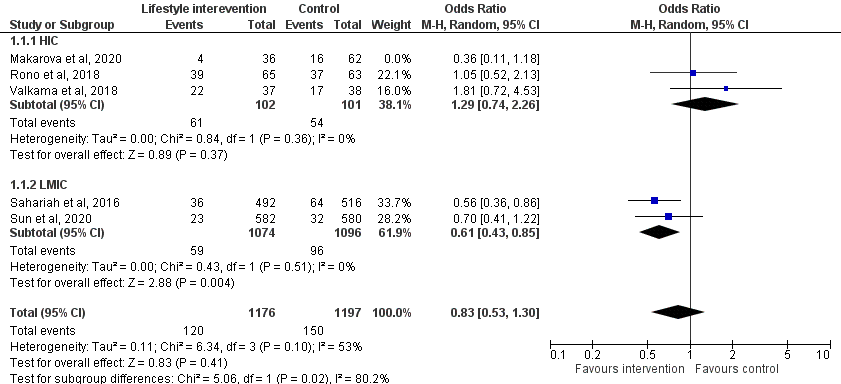
Supplementary figure 3a) Leave one out plots for RCTs**

**
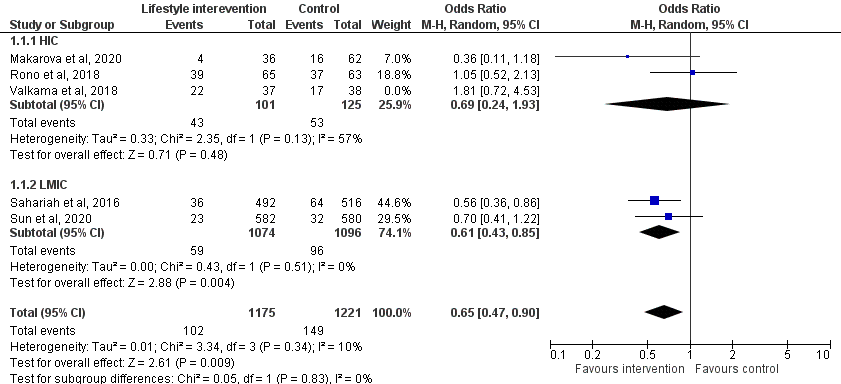
**

**
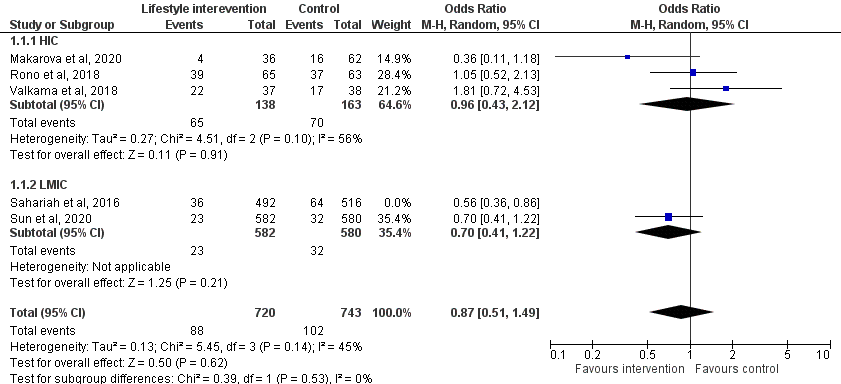
**

**
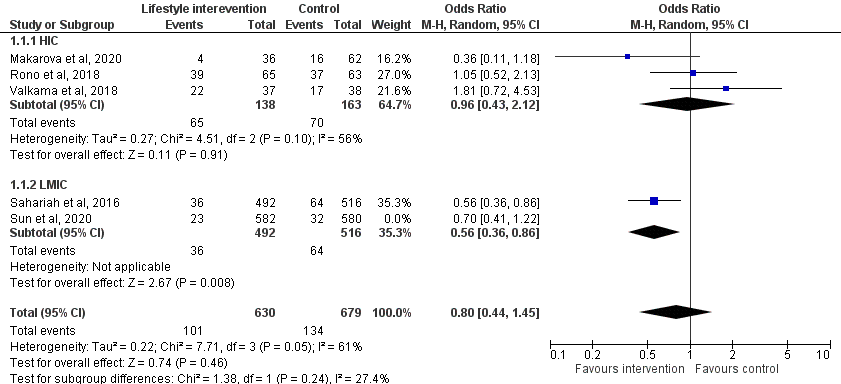
Supplementary figure 3b) Leave one out plots for cohort studies based on Physical activity**

**
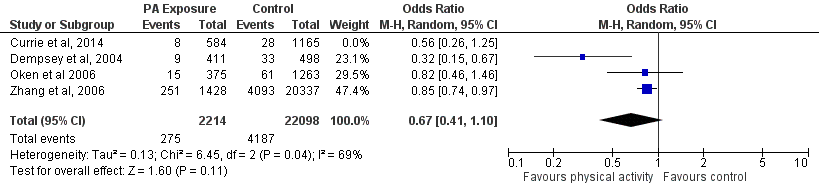
**

**
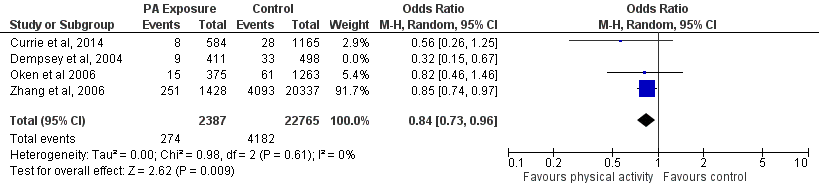

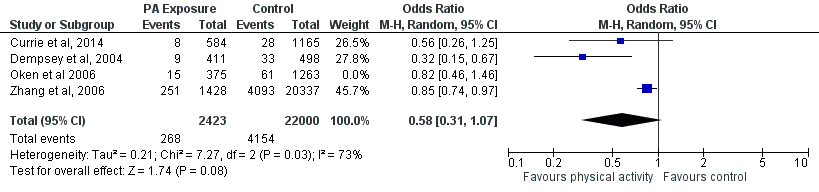

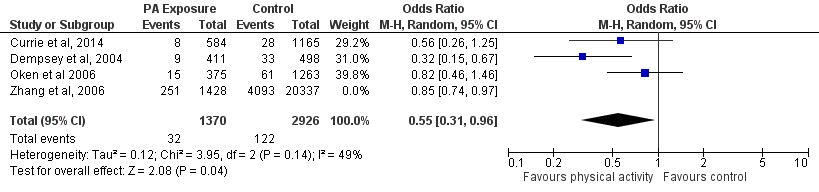
**

**Supplementary figure 3c) Leave one out plots for cohort studies based on Low Carbohydrate/low sugar diet**

**
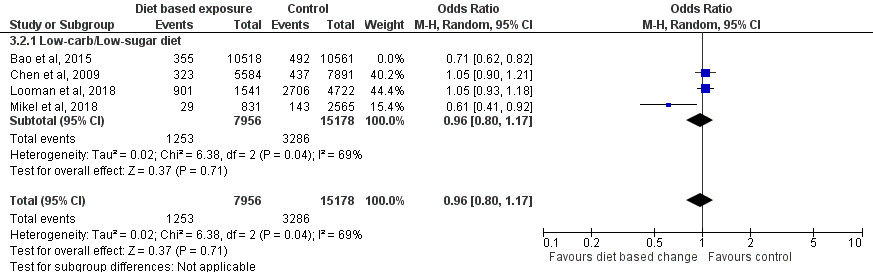
**

**
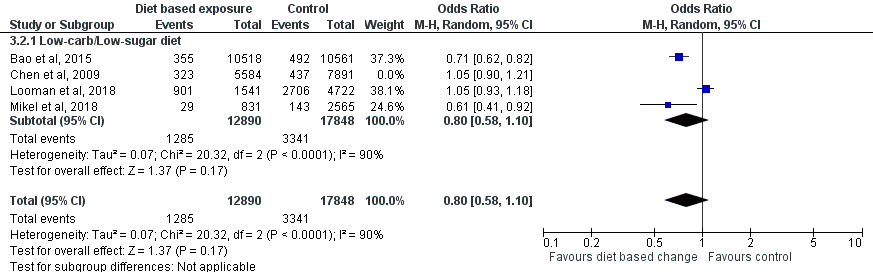
**

**
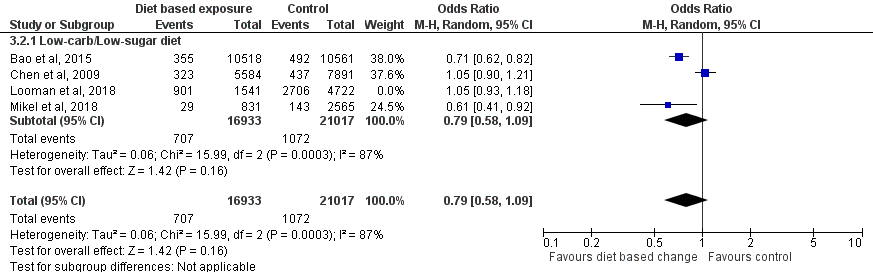
**

**
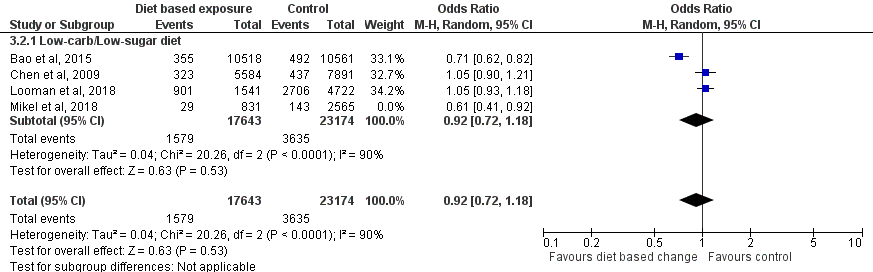
**

**Supplementary figure 3d) Leave one out plots for cohort studies based on Diet Scores**

**
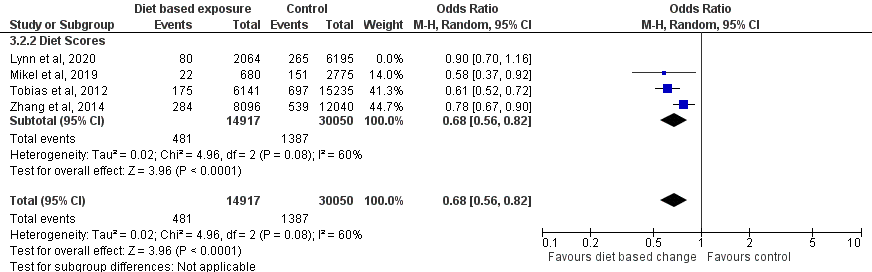
**

**
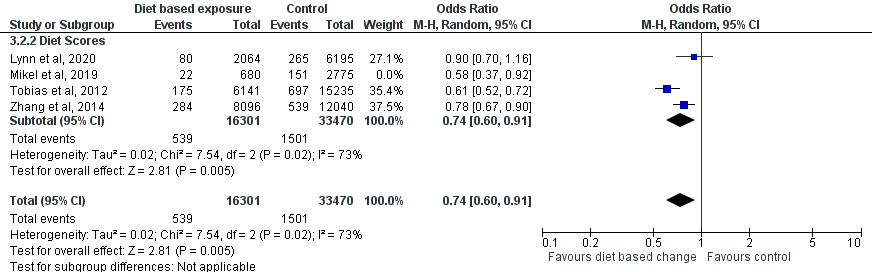
**

**
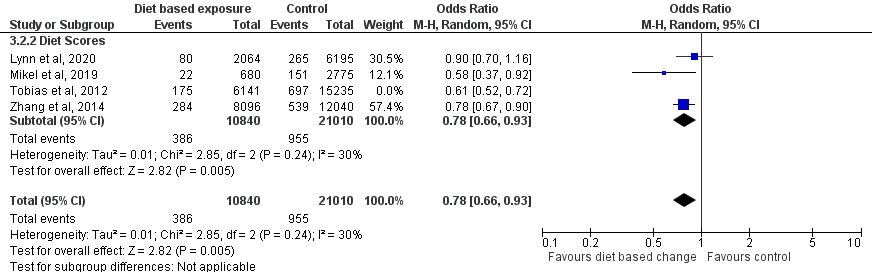
**

**
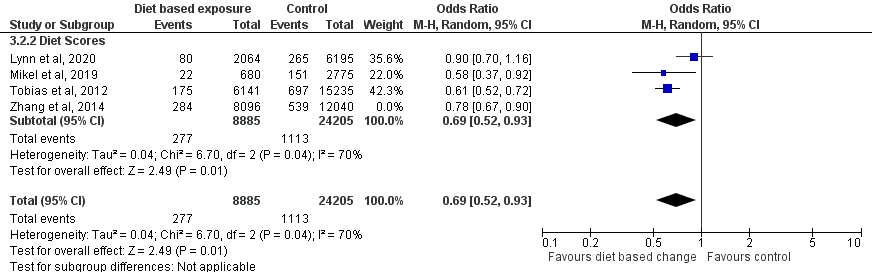
**
